# Soil CO_2_ Efflux in a Mixed Pine-Oak Forest in Valsaín (Central Spain)

**DOI:** 10.1100/tsw.2007.7

**Published:** 2007-03-21

**Authors:** Rosa Inclán, Daniel De la Torre, Marta Benito, Agustín Rubio

**Affiliations:** ^1^ Ecotoxicology of Air Pollution. Ciemat, ed 70. Avda., Complutense 22, Madrid-28040, Spain; ^2^ Dept. Edafología, Universidad Politécnica de Madrid, Ciudad Universitaria, Madrid, Spain; ^3^ Dept. Silvopascicultura, Universidad Politécnica, Madrid, Spain

**Keywords:** particulate organic matter, soil organic matter quality, ecotone, Quercus pyrenaica, Pinus sylvestris, forest ecosystems, climate change, soil CO^2^ efflux, soil water content, soil temperature

## Abstract

Soil-surface CO_2_ efflux and its spatial and temporal variation were investigated in a southern Mediterranean, mixed pine-oak forest ecosystem on the northern slopes of the Sierra de Guadarrama in Spain from February 2006 to July 2006. Measurements of soil CO_2_ efflux, soil temperatures, and moisture were conducted in nine 1963-m^2^ sampling plots distributed in a gradient around the ecotone between *Pinus sylvestris* L. and *Quercus pyrenaica* Lam. forest stands. Total soil organic matter, Walkey-Black C, particulate organic matter, organic matter fraction below 53 μm, total soil nitrogen content, total soil organic carbon content, and pH were also measured under three representative mature oak, pine, and mixed pine-oak forest stands. Soil respiration showed a typical seasonal pattern with minimums in winter and summer, and maximums in spring, more pronounced in oak and oak-pine stands. Soil respiration values were highest in pine stands during winter and in oak stands during spring and summer.

Soil respiration was highly correlated with soil temperatures in oak and pine-oak stands when soil moisture was above a drought threshold of 15%. Below this threshold value, soil moisture was a good predictor of soil respiration in pine stands. Greater soil organic matter, particulate organic matter, Walkey-Black C, total organic C, and total N content in pine compared to oak sites potentially contributed to the greater total soil CO_2_ efflux in these stands during the winter. Furthermore, opposing trends in the organic matter fraction below 53 μm and soil respiration between plots suggest that in oak stands, the C forms are less affected by possible changes in use. The effects of soil properties on soil respiration were masked by differences in soil temperature and moisture during the rest of the year. Understanding the spatial and temporal variation even within small geographic areas is essential to assess C budgets at ecosystem level accurately. Thus, this study bears important implications for the study of large-scale ecosystem dynamics, particularly in response to climatic change.

## INTRODUCTION

Carbon (C) enters terrestrial ecosystems through photosynthesis and is returned through respiration. In forests, total ecosystem respiration tends to be dominated by soil respiration (SR)[1], accounting for 60–80% of total ecosystem respiration and affecting the interannual variability of net ecosystem production, particularly in Mediterranean forest ecosystems. Although these ecosystems may not be very relevant in terms of the C that they store, they are highly vulnerable to climate change. Such changes may induce a loss of biodiversity, especially where populations are isolated, in marginal sites, ecotones, and system transition zones within the distribution area of the species. In this regard, a lack of natural regeneration in Scots pine (*Pinus sylvestris* L.) stands and the gradual replacement of this species by the broadleaved *Quercus pyrenaica* Lam, has been detected in the southwestern part of Spain over the last century. Shifts in the relative abundance of species change the patterns of C flow through ecosystems and affect both the amount and residence time of C on land[2]. Estimates of future changes in $CO_2$ depend strongly on the feedback of terrestrial ecosystems to climate change. Therefore, reliable estimates of SR are required in order to represent forest-atmosphere interactions in global modeling studies.

General models are often based on single variables, such as mean annual soil temperature (ST)[3,4,5,6]. In Mediterranean ecosystems, SR fluxes are highly sensitive to soil moisture (SM). Low or high soil water content may limit SR by either drought stress to microbial communities and root activities, or by limiting oxygen availability due to reduced air diffusion for decomposition and root maintenance and growth[3,4]. SR is also reportedly influenced by site fertility[7]. The amount and type of soil organic matter (SOM) probably affects SR, given that the relationship between decomposition rates and temperature may be different for different types of SOM. In the SOM quality studies, the particulate organic matter (POM), which refers to the 53- to 2,000-μm fraction of the organic matter, represents the labile fraction of SOM, while the organic matter fraction below 53 μm (OM < 53) refers to the mineral-associated fraction, which is more stabilized and presents longer turnover times[8]. It is well known that tree species and nutrient availability strongly affect the decomposition of litter; consequently, tree species and specific soil factors could influence SR.

The main objectives of the work presented here were (1) to investigate the temporal and spatial variability of SR in the southern location of a mixed *P. sylvestris* L. and *Q. pyrenaica* Lam. forest in the central mountain range of Spain and (2) to analyze the effects of environmental conditions (ST, SM) and other soil characteristics (Walkey-Black C, total SOM, POM, OM < 53, total organic carbon, total nitrogen content, C/N, pH) on soil $CO_2$ efflux.

## MATERIAL AND METHODS

### Study Site

The study took place in central Spain (Valsaín, Segovia), (40º 51´N, 4º3´W), on the northern face of the Sierra de Guadarrama. The altitude ranges between 1,270 and 1,360 m a.s.l. The total area of Valsaín Forest is 10.672 ha, covered mainly by Scots pine (*P. sylvestris* L.). Other species present include oak (*Q. pyrenaica* Lam.) and montane broom (*Cytisus purgans* [L.] Boiss.), along with small areas of Holm oak (*Q. ilex* subsp. *ballota* [Desf.] Samp.) and riparian forest. The herbaceous layer is largely made up of grasses that develop in spring, dry off in summer, and regrow to some extent after the first autumn rains. The National Parks Agency and the “Montes de Valsaín” Centre are the organizations responsible for its management.

The climate is nemoro-mediterranean, with an annual average rainfall of 1,600–1,400 mm, falling to a minimum in summer (15.1 mm in July). Mean temperatures in January and July are 1.5–2.7º and 19.7–20.3º, respectively. The mean maximum temperature is 26.2º and the mean minimum temperature is –1ºC. The rainfall distribution is irregular, with a drought period in summer of approximately 2 months.

Geologically, medium to large grain granite is predominant. Soils are classified as Humic Cambisols or Typic Haplumbrepts.

The management methods employed in this forest favor the compatibility of forest harvesting and cattle grazing, and promote the continuation of traditional activities alongside the need for habitat preservation and improvement, while also providing for the requirements of the human population. The pinewood stocks comprise 3,234.572 $m^3$, with an annual felling of 36,048 $m^3$. Thinning in the oak coppice is carried out by cutting between 15 and 20% of the basal area. This is done by felling the dead and dominated trees. The annual yield is about 501 $m^3$.

## Experimental Design

A transect around the ecotone between *P. sylvestris* L. and *Q. pyrenaica* Lam. forest stands was established in 2005. Three representative forest plots with a radius of 25 m were selected, under *Q. pyrenaica* (Q), *P. sylvestris* (P), and *Q. pyrenaica-P. sylvestris* (Q-P). We used three replicates per stand. In each sampling zone, 12 soil sampling sites were chosen, including four under Q stands, four under P stands, and four under Q-P stands for soil determinations.

Within the plots, 20 PVC collars (10 cm in diameter and 4.5 cm in length) per stand were inserted into the soil to an average depth of 2.5 cm to measure total soil $CO_2$ fluxes. Collars were proportionally distributed over the plot, and left in place throughout the course of the experiment.

The mean diameter, mean height, and basal area for each tree were calculated using data collected during the tree survey performed at the beginning of the study, in spring 2005. All the trees within each plot were measured. A summary of the site characteristics is given in Table 1.

**TABLE 1**
**Some Characteristics of the Study Sites at the Beginning of the Study, May 2005**

| Site | Geographical Location (UTM) | | *Q. pyrenaica* | | | | *P. sylvestris* | | | |
|---|---|---|---|---|---|---|---|---|---|---|
| | | | Mean Diameter (cm) | Mean Height (m) | Basal Area ($m^2$/ha) | Stem Density (trees/ha) | Mean Diameter (cm) | Mean Height (m) | Basal Area ($m^2$/ha) | Stem Density (trees/ha) |
| | X | Y | | | | | | | | |
| Q | 410040 | 4524475 | 14.5 | 9.7 | 17.24 | 932 | — | — | — | 0 |
| Q-P | 410158 | 4523820 | 12.8 | 9.4 | 4.39 | 331 | 48.2 | 18.8 | 26.20 | 132 |
| P | 409968 | 4523274 | — | — | — | 0 | 31.5 | 19.0 | 64.31 | 657 |

## Soil $CO_2$ Efflux Measurements

Soil $CO_2$ fluxes were measured randomly in each forest plot on a monthly basis over the year 2006, using a closed dynamic system LI-6400 coupled to a LI-6400-9 soil chamber (LI-COR Inc, Lincoln, NE). The measurement of soil $CO_2$ efflux consisted of placing the chamber on the collar, scrubbing the $CO_2$ to subambient levels, and measuring the flux rate as it rose from 10 ppm below to 10 ppm above the atmospheric value. Soil $CO_2$ efflux sampling was not performed on days following a rain event to avoid an overestimation of the efflux due to $CO_2$ displacement from soil pores[3]. Measurements were made between 10:00 a.m. and 16:00 p.m. to minimize the diurnal variation in SR.

## Soil Temperature and Moisture Measurements

ST and SM contents were measured concurrently with soil $CO_2$ efflux using a thermocouple sensor (Omega Engineering, Stamford, CT) and a time-domain reflectometry system (TRIMEGM, IMKO GmbH, Ettlingen, Germany) located next to the collars where soil efflux measurements were taken, at a depth of 10 cm.

## Chemical Properties of Soil

In October 2005, four composite soil samples were taken from each plot at a depth of 15 cm. Soil samples were air dried, sieved through a 2-mm sieve, ground, and homogenized to determine chemical properties. The readily oxidizable organic carbon (COX) was measured following Walkley-Black's method[9]. The total SOM was estimated by a modification of the "loss on ignition" method (LOI)[9]. The separation of the POM fraction was made following[10]. The OM content of fractions 53–2,000 μm and OM < 53 was estimated by LOI. Soil pH was measured with a glass electrode using a sample-to-water ratio of 1:2.5. Total nitrogen content (N) was determined by Kjeldahl digestion[11]. Total organic carbon (TOC) was estimated by the dry combustion method at 540ºC[12]. All the analyses were made with two replicates. Soil parameters are given in g/kg.

## Statistical Analysis

All statistical analyses utilized Statistica v 6.0 (Statsoft, Inc., USA). Data sets were tested for normal distribution and homogeneous variance by the Kolmogorov-Smirnov and Levene's tests, respectively. One-way analysis of variance was performed to analyze the differences in soil $CO_2$ efflux and various soil properties between stands. Post-hoc comparisons were tested using Tukey's HSD test, calculated at the 5% level. Linear regressions were performed to model the temperature and water dependency of SR from each stand.

## RESULTS AND CONCLUSIONS

The rates of SR measured at Valsaín were comparable to those reported in previous studies on sites exposed to drought[13,14]. As a general trend, SR showed a typical seasonal pattern with minimums in winter and summer, and maximums in spring (Fig. 1). This pattern is more pronounced in Q and Q-P stands. The analysis of the monthly values obtained at the different stands showed that SR values were highest in P stands during winter, and in Q stands during spring and summer. In Q-P stands, SR rates were not significantly different from each other. In winter, the SR rates in Q-P stands were similar to those in P stands, and in summer, they were similar to those in Q stands. During March, the efflux rates were not significantly different between treatments.

Both ST and SM content varied according to the season. Maximum ST coincided with minimum SM content during the summer, and minimum ST was recorded in winter when SM was highest (Fig. 1). Temperatures over spring and early summer were highest ($p < 0.05$) in the Q stands, followed by the Q-P and P stands. No significant difference was observed among the stands in winter. SM content over spring and early summer was highest in the Q stands. SM was lower ($p < 0.05$) in P stands during summer and in Q stands during winter.

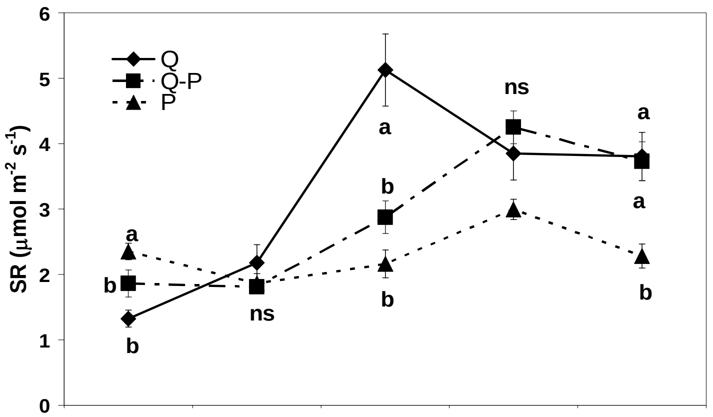

A

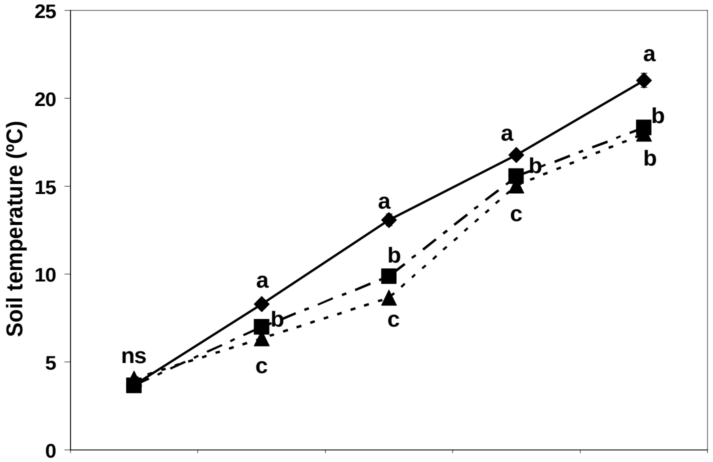

B

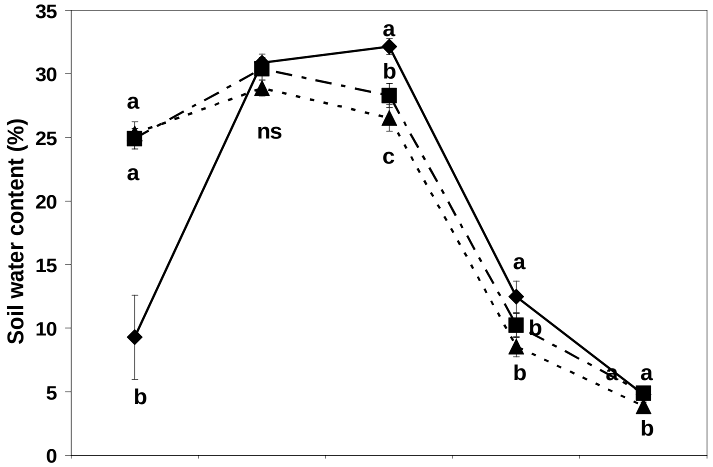

C

**FIGURE 1**. Seasonal variation of (A) soil respiration, (B) soil temperature, (C) soil moisture, and (D) monthly precipitation in the stands: P (*P. sylvestris* L), Q (*Q. pyrenaica* Lam), Q-P (*Quercus-Pinus*) from February 2006 to July 2006 (no data are available for January when snow covered the ground or in April). Each value represents the mean of 20 measurements at each stand; bars indicate ± SE. Different letters indicate significant differences between stands at $p < 0.05$; ns = not significant.

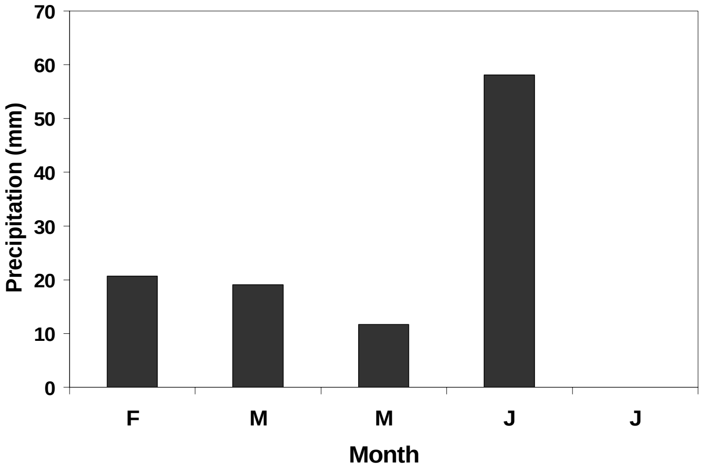

**FIGURE 1D**

The SOM, POM, TOC (Fig. 2), and C/N (Table 2) were higher in P stands than in Q stands. No significant difference was detected among the Q and Q-P stands. With respect to COX and OM < 53 (Fig. 2), the higher values were found in the P stands, followed by Q-P and Q stands, respectively. The higher SOM values under *Pinus* stands is a result of higher % POM and lower % OM < 53 (Table 2). The much lower bioavailability of *Pinus* litter as compared to that of *Quercus*, and the reduced microbial activity in the acid soil (Table 2) probably favored POM accumulation in soil. The higher C/N in the P stands indicated a higher degree of humidification in SOM. With respect to % OM < 53, the higher value in the Q (63.37%) vs. P (50%) suggests that in *Quercus* stands, the C forms are less affected by possible changes in use. A significant correlation between POM and COX in Q stands (r = 0.937, $p < 0.05$) indicates that POM is primarily constituted by the most labile forms of SOM. In soils under P forest, POM is not only constituted by the most labile C forms, but also by recalcitrant C forms.

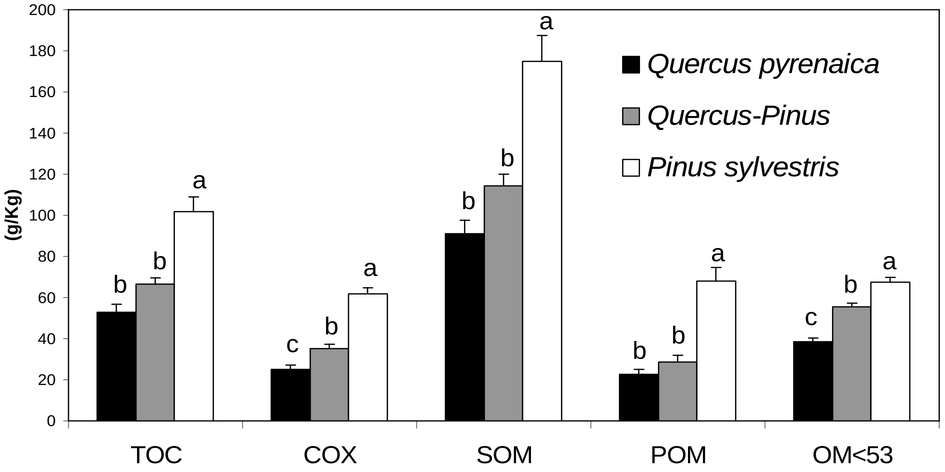

**FIGURE 2.** Means of total soil organic carbon (TOC), Walkey-Black C (COX), total soil organic matter (SOM), particulate organic matter (POM), organic matter fraction below 53 µm (OM < 53) in the mineral soil at 0- to 15-cm depths in the *Quercus*, *Pinus,* and *Quercus-Pinus* stands at Valsaín forest. Different letters denote significant differences between plots ($p < 0.05$). Error bars represent ± SE (n = 4).

**TABLE 2**
**A-Horizon Characteristics (0–15 cm) of Investigated Sites in Valsaín**

| Site | pH $_{(H20)}$ | C/N | % POM $_{(a)}$ | % OM < 53 $_{(b)}$ |
|---|---|---|---|---|
| Q | 5.81 ± 0.12 a | 10.04 ± 0.3 b | 36.62 ± 1.60 b | 63.37 ± 1.60 a |
| Q-P | 5.44 ± 0.06 b | 10.86 ± 0.3 b | 33.09 ± 2.22 b | 66.90 ± 2.22 a |
| P | 5.47 ± 0.03 b | 15.93 ± 0.4 a | 49.44 ± 1.91 a | 50.55 ± 1.91 b |

*Note:* Means with different letters within site are significantly different using Tukey's HSD at the 0.05 level.

(a) % POM = POM/(POM + OM < 53).

(b) % OM < 53 = OM < 53/(POM + OM < 53).

We investigated the relationship between SR and ST when soil water was both above and below a drought threshold of 15%. An important fraction of the temporal variation in SR was explained by ST in Q stands (Fig. 3a). STs displayed the strongest relationship with soil $CO_2$ efflux when SM was above the threshold value of 15% in Q-P stands (Fig. 3b); below this threshold value, soil moisture was a good predictor of soil respiration in P stands (Fig. 3c). A similar threshold value for SM has been found in other drought-stressed regions[3,13,14]. The findings of this study agree with those of previous studies carried out in the Mediterranean region, in that low soil water content limits the response of SR to ST.

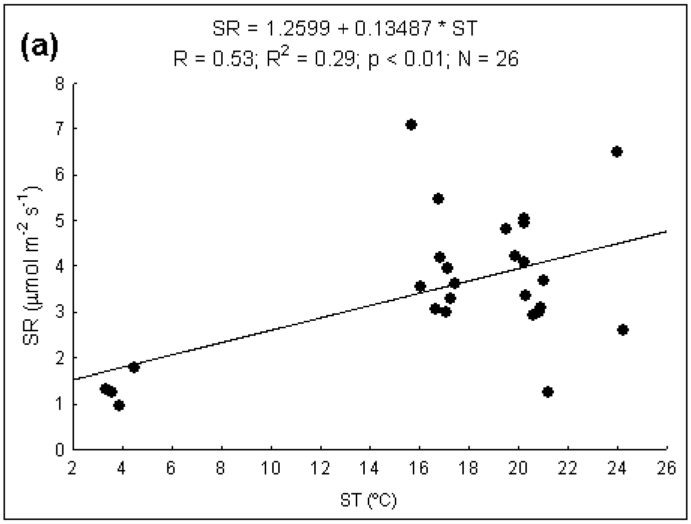

**FIGURE 3.** The relationships between soil respiration, soil temperature, and soil volumetric water content during the experimental period in the *Quercus* (a), *Quercus-Pinus* (b), and *Pinus* (c) stands at Valsaín forest.

Soil parameters SOM, POM, TOC, and C/N were positively related with SR, whereas the pH value was negatively related with SR in P stands in winter. The results of this study, which reveal increased SR in response to the increase in several soil quality parameters in winter, are consistent with studies[13,15]. However, no clear relationship was detected between spatial variation in SR and the heterogeneity of soil properties during the rest of the months of our study. This agrees with others findings and suggests that the temporal variability of ST and SM has a greater effect on SR than the spatial variability[14].

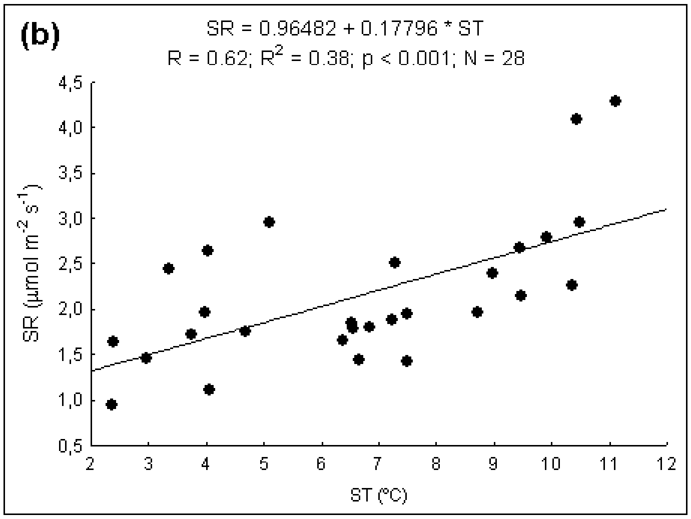

**FIGURE 3b**

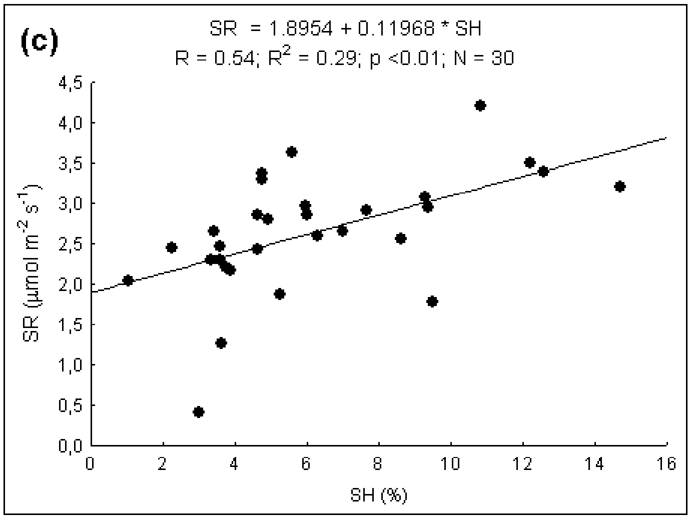

**FIGURE 3c**

In combination, our results suggests that, even within a relatively small geographical area, variation in soil properties, temperature, and moisture can influence soil $CO_2$ efflux in mixed pine-oak forests located in the southwest. This implies that changes in soil properties, temperature, and moisture over small scales need to be considered when modeling landscape-level $CO_2$ efflux.

The main conclusions that can be drawn from this study in a mixed pine-oak forest under Mediterranean climate conditions are as follows:

- The seasonal variation of soil $CO_2$ efflux recorded in our study confirms that SR is controlled by ST and SM as stated in previous studies undertaken in Mediterranean conditions.
- We observed spatial variation in SR related to the heterogeneity of soil properties and the temporal variability of ST and SM in winter. However, the effects of soil properties on SR were masked by differences in ST and SM during the rest of the year.

The results from this experiment indicate that projected changes in precipitation patterns may have much larger effects on SR and, in turn, on the C balance of southwest Mediterranean Scots pine stands. These results suggest that the application of standard models to estimate SR for regional and local geographic areas may not be adequate unless other factors are considered in addition to ST. Thus, changes in soil properties at small scales and the availability of SM need to be considered when modeling $CO_2$ efflux in drought-prone sites. Further research over longer periods is required to validate and assess the importance of site productivity and stand structure on SR.

## ACKNOWLEDGMENTS

This research was conducted in the framework of Spanish HU2005-0023, AGL2004-01941, and CGL 2006-02922/CLI projects, and the COST 639 *(BurnOut)*.
